# Health and Revenue

**Published:** 1901-03-23

**Authors:** 


### The Hospital

A Journal of

The Medical Sciences and Hospital Administration.

Vol. XXIX.—No. 756.] Edited by Sir Henry Burdett, K.C.B., and Solomon C. Smith, M.D., M.R.C.P. [March 23, 1901.

### Health and Revenue.

Few things are more certain than that, within definite limits, the consumption of articles of luxury, and even of some which occupy a borderland between luxury and necessity, may be modified by the incidence of taxation. Perhaps the best illustration is furnished by the change in the national taste as regards wine which was produced by the Methuen Treaty of 1703, under which the wines of Portugal were admitted at one-third of the duty charged upon those of France, and gradually superseded them at almost all tables, thus prevailing not only over long-established custom, but also over the still more powerful dictates of fashion. The latter were sufficiently strong to form a distinct basis of opposition to Marlborough, on the part of the drinkers of French wine, in the early days of the reign of Queen Anne, but they soon yielded to the influence of economy. Everyone remembers the epigram which was then, or soon after, current in relation to the effect of the change upon the national spirit of Scotland:—

Firm and erect the Caledonian stood,
Tender his mutton, and his claret good;
Until at length "Drink port," the tempter cried,
He drank the poison, and his spirit died.

As an unpublished appendix to this we may mention having heard from a venerable Scottish lady that, in her girlhood, Walter Scott's friend, William Erskine, afterwards Lord Kinedder, dined with her father and grumbled at the absence of claret from the table. When the gentlemen, more or less "fatigued with drinking," at length came to the drawing-room, our informant was called upon to sing, and selected the sweet old ballad, "My Jo Janet," in the course of which she mischievously interpolated an extempore verse of her own:—

Drink the port, the claret's dear,
My Jo Erskine!
Ye'll get drunk, never fear,
My Jo Erskine!

The records of the Exchequer are full of analogous instances of the effects of taxation, and the experience thus accumulated may often be usefully applied in the distribution of burdens over different sections of the community; subject, of course, to the well-established principle that high duties may so far diminish consumption as not to produce an appreciable increase of revenue, and that it is generally inexpedient to tax either the necessaries of life or the raw materials of any important industry. Even when these fundamental principles are strictly acted upon, there will remain a considerable field of action in which the ingenuity of the Chancellor of the Exchequer may be wisely and profitably exercised.

The present holder of that office will to all appearance soon be called upon to impose new burdens on the community; burdens light indeed when compared with those of less favoured nations, but heavy by contrast with those to which we are accustomed. Is it too much to expect that consideration may be given to the degree in which habits affecting the national health are capable of being influenced by financial considerations? The recent inquiries into arsenical poisoning by beer have brought to light a frequent consumption, by a single individual, of as much as two or three gallons a day of the incriminated beverage; and a duty, sufficient to be prohibitory of excess of this description, would not interfere with the "modest pint" of the moderate consumer. Quack medicines, again, are among the agencies most injurious to the public health. The chief cause of hæmorrhoidal affections, and of all the miseries which spring from them, is unquestionably the enormous consumption of the cheap and coarse purgatives which constitute the bases of the "family" or other pills the titles of which are so conspicuous in omnibuses and on hoardings. The returns of the present taxation of these things is said to show a public expenditure upon them of about three millions a year; and no one could complain if spherules described by their owners as being "worth a guinea a box," were rendered inaccessible at the price of thirteenpence halfpenny at which they are now retailed. Surely one eighth of their alleged value would not be an exorbitant charge for them; and, even if diminished consumption were attended by a corresponding diminution of the fees now accruing to gentlemen engaged in rectal surgery, the community at large would still be gainers. Tobacco is a tender subject. The Chancellor of the Exchequer has already said that he does not see his way to special taxation of the too seductive and too frequent cigarette; so that small boys are not likely to be checked financially in their present practice of muddling away their poor little brains by continual indulgence in a narcotic which is often injurious even to strong adults. Financiers will no doubt tell us that taxation should rest upon broad lines and simple principles; but it may be remembered that Sir Robert Peel once established a new tariff ranging over an enormous number of articles, many of which were totally unknown, even by name, to the average man. A similar course would enable his successor to promote the letting alone of many things the employment of which is certainly of no advantage to the public.

